# Milia En Plaque Associated With Prayer-Related Frictional Changes

**DOI:** 10.7759/cureus.85507

**Published:** 2025-06-07

**Authors:** Moosa A Hira, Fahad S Siddiqui, Robert Moore, Zain U Syed

**Affiliations:** 1 Dermatology, Skin Care Specialty Physicians, Lutherville, USA; 2 Dermatology, Kansas City University-Graduate Medical Education Consortium/Advanced Dermatology and Cosmetic Surgery, Maitland, USA; 3 Pathology, HCT Pathology, Baltimore, USA

**Keywords:** dermatology and dermatologic surgery, friction-induced dermatosis, milia en plaque, muslim prayer, prayer-related dermatoses, surgical skin excision

## Abstract

Milia en plaque (MEP) is an uncommon dermatologic condition characterized by grouped milia on an erythematous plaque. Its underlying cause remains unclear, though various internal and external factors may contribute. We present the first reported case of MEP linked to prayer-related frictional changes. A 67-year-old South Asian male developed a well-demarcated plaque with agminated milia on his forehead at the site of chronic mechanical friction from prayer-related prostration. Histopathology revealed cystically dilated follicles and inclusion cysts without atypia. Surgical excision led to complete resolution without recurrence at two months, with an excellent cosmetic outcome. This case highlights not only a novel trigger for MEP but also suggests that surgical excision may be an effective treatment option for localized lesions, offering favorable aesthetic results. Awareness of mechanical factors in MEP pathogenesis may guide prevention and individualized management strategies.

## Introduction

Prayer-related dermatoses (PRD) encompass various skin changes associated with prayer rituals and, in Muslim patients, commonly associated with frictional or pressure-related changes during prayer, often affecting the forehead [1]. Milia en plaque (MEP) is a rare condition characterized by grouped milia on an erythematous, plaque-like base [2]. While the exact etiology remains unclear, associations with autoimmune conditions and genetic predisposition have been noted [3]. Most cases occur in the periauricular region; however, other areas may be involved. To our knowledge, this is the first reported case of MEP linked to prayer-related frictional changes, with successful resolution following surgical excision.

## Case presentation

**Figure 1:**
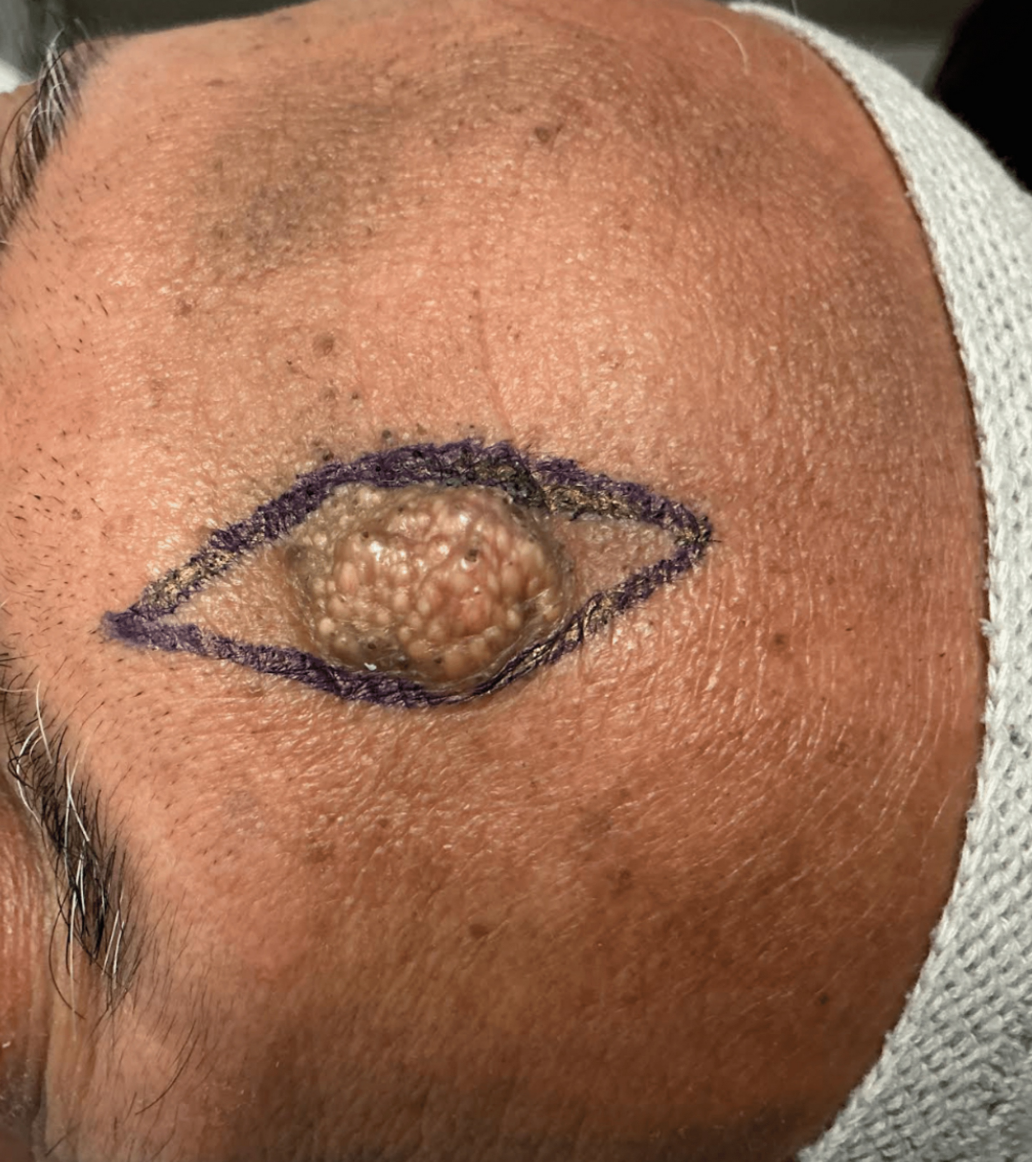
Preoperative frontal appearance of the lesion on the left forehead with planned elliptical excision. On the right forehead, mild hyperpigmentation and lichenification are noted.

A 67-year-old South Asian male with a history of gout and hypertension presented with an asymptomatic lesion on his forehead, seeking removal for cosmetic reasons (Figure 1). He first noticed the lesion five years ago, with gradual enlargement over that time. He also reported performing prayer five times a day, each involving several minutes of prostration. Examination revealed a 1.8 × 1.5 cm well-demarcated soft plaque with agminated yellow-white papules on an ill-defined hyperpigmented lichenified base on the left forehead. A similar hyperpigmented, lichenified area measuring 1.6 × 1.9 was present on the contralateral forehead but without associated plaque.

**Figure 2:**
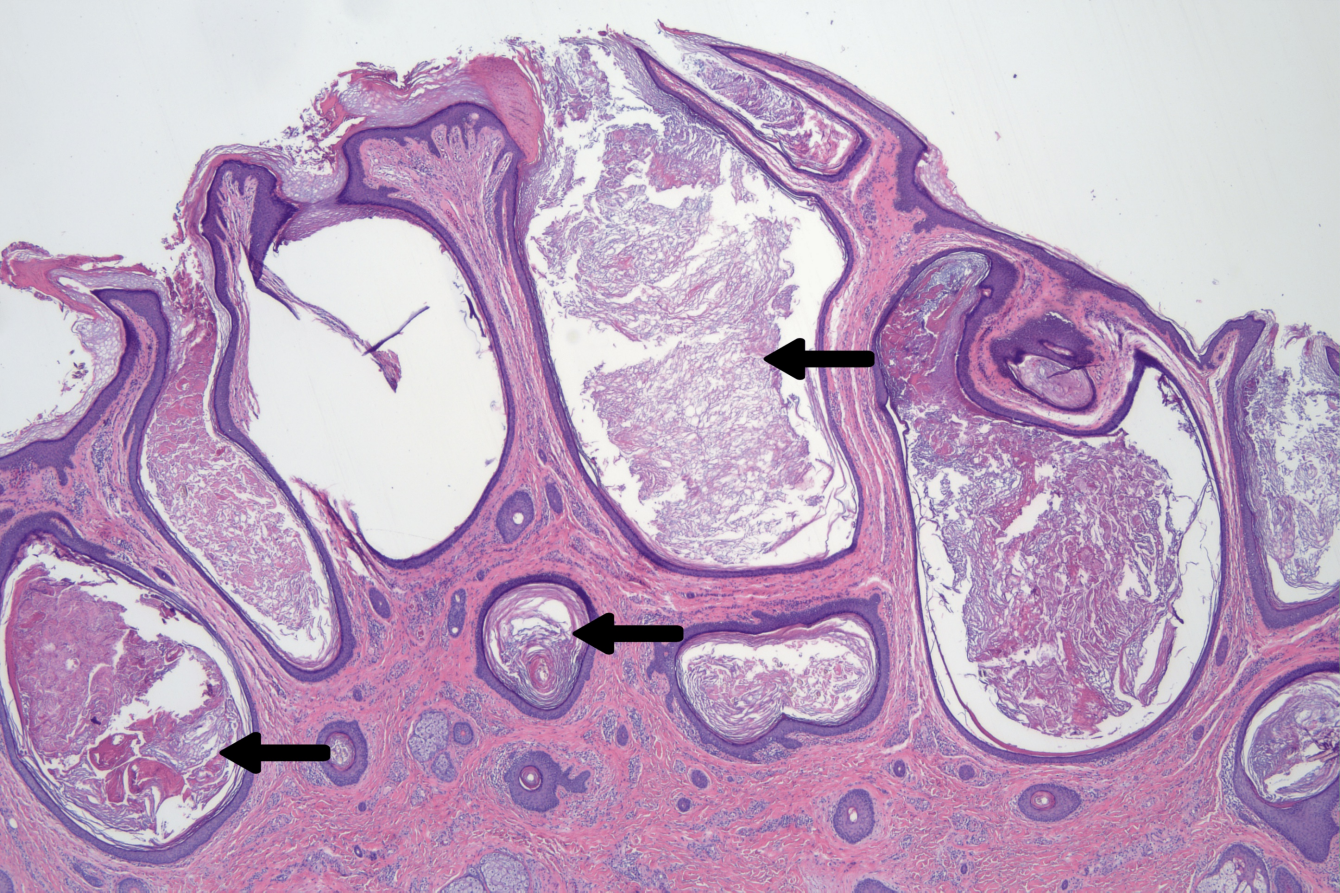
Hematoxylin and eosin (H&E) stained section. Plaque-like cystic proliferation of dilated follicles and inclusion cysts (arrows).

**Figure 3:**
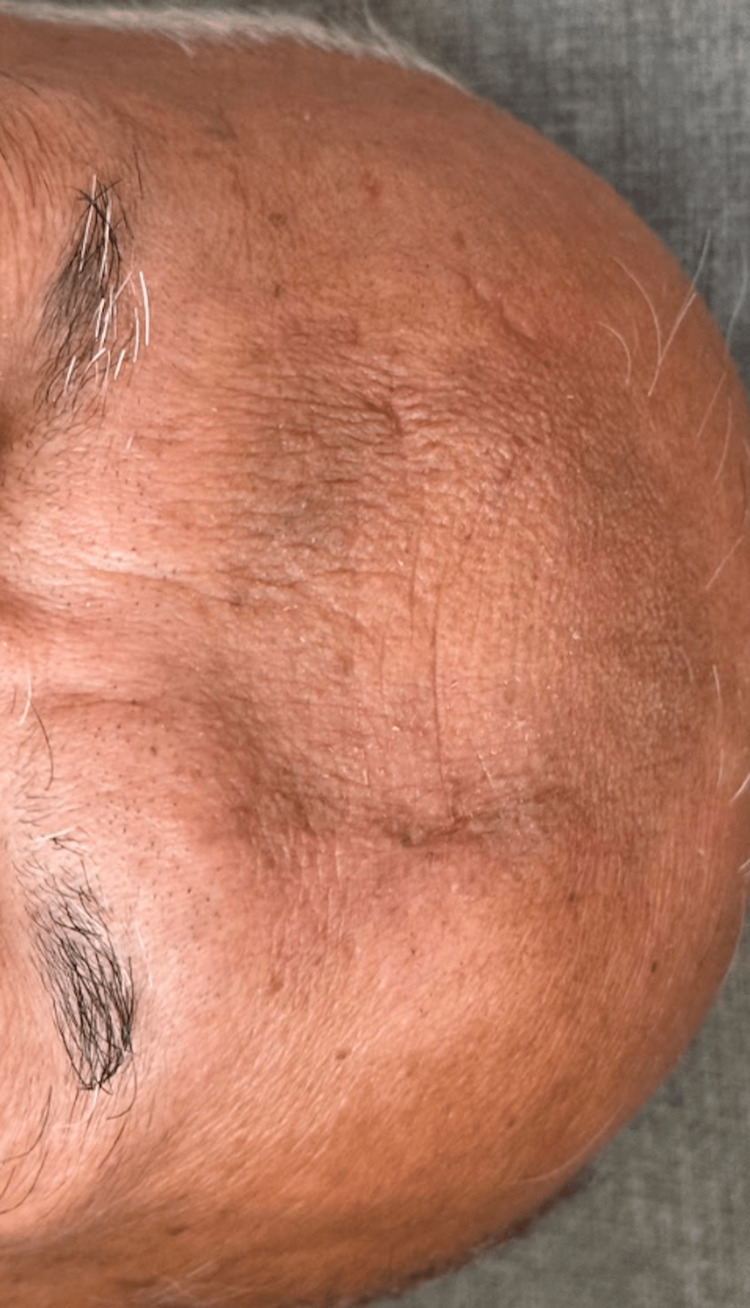
Well-healed scar line at two months without evidence of recurrence.

Elliptical excision of the plaque was performed and closed with 5-0 poliglecaprone buried horizontal mattress sutures and 6-0 plain gut running sutures. Histopathological analysis revealed a cystic proliferation of dilated follicles and inclusion cysts without evidence of cytologic atypia or malignancy (Figure 2). The differential diagnosis included Favre-Racouchot syndrome, nevus comedonicus, and MEP. Considering the lesion’s circumscribed nature, lack of solar elastosis, and acute growth, MEP was the most likely diagnosis. At a two-month follow-up, the lesion had resolved without recurrence (Figure 3). The patient was scheduled for periodic follow-up appointments to monitor for recurrence.

## Discussion

MEP is a rare, benign condition characterized by multiple small milia on an erythematous plaque, with fewer than 70 cases reported [4]. Although typically asymptomatic, MEP may present with cosmetic concerns. The etiology of MEP remains unclear. It is often idiopathic but has been associated with genodermatoses (e.g., epidermolysis bullosa, porphyria cutanea tarda), bullous diseases (e.g., bullous pemphigoid, lupus erythematosus), medications (e.g., cyclosporine, penicillamine, corticosteroids), trauma, and dermatologic procedures [4,5]. Despite these associations, most cases lack a clear predisposing factor.

MEP primarily affects the head and neck, especially the periauricular and periorbital regions [5]. Other reported sites include the nasal bridge, supraclavicular area, cheek, chin, and lower limbs [6]. Histologically, MEP consists of keratin-filled epidermoid cysts within the dermis, lined by stratified squamous epithelium, sometimes containing vellus hairs. Inflammatory infiltrates vary from mild lymphocytic infiltration to dense mononuclear infiltrates, particularly in cases linked to lupus erythematosus [6].

Treatment options for MEP vary due to its rarity. First-line therapies include topical retinoids, which may benefit superficial lesions [4]. Simple extraction has often resulted in recurrence. Advanced treatments include systemic retinoids (e.g., etretinate), oral minocycline, cryotherapy, electrodesiccation, and CO2 or erbium: YAG laser therapy [5,6]. Photodynamic therapy has shown partial success but is costly [4]. In our case, surgical excision appeared to be an effective option, with a favorable cosmetic outcome and no recurrence at two months post-op.

To our knowledge, this is the first reported case of MEP arising on the forehead, most probably caused by chronic friction from prayer-related prostration. "Prayer marks" commonly appear as hyperpigmented, lichenified plaques on the forehead, knees, and feet due to mechanical trauma [1]. However, no prior reports have linked MEP to prayer-induced friction or lichenification. Given MEP’s potential triggers, repetitive friction may have contributed to MEP formation in this patient. This novel association expands the understanding of MEP’s etiology and highlights the role of mechanical friction in its pathogenesis. For cases where MEP results from chronic friction, patient education on mechanical factors and preventive strategies, such as softer prayer surfaces, may be beneficial. Individualized treatment should consider lesion depth, inflammation, and patient preferences.

## Conclusions

This case highlights a novel association between prayer-related frictional trauma and the development of MEP. Recognition of mechanical factors as potential triggers for MEP may aid in the prevention and management of the condition. Surgical excision proved to be an effective treatment option in our patient, offering complete resolution and an excellent cosmetic outcome at two months. Further reports may help elucidate the role of trauma in MEP pathogenesis and guide optimal therapeutic strategies.
